# Racial and Ethnic Disparities in Quality Measures for People With Alzheimer’s Disease and Related Dementias

**DOI:** 10.1093/geroni/igaa057.2306

**Published:** 2020-12-16

**Authors:** Maricruz Rivera-Hernandez, Amit Kumar

**Affiliations:** 1 Brown University School of Public Health, Providence, Rhode Island, United States; 2 Northern Arizona University, Flagstaff, Arizona, United States

## Abstract

Over 50% of nursing home residents have ADRD. Prior research has shown strong evidence that, compare to non-Hispanic White, African-Americans are more likely to admitted in low performing nursing homes characterized by severe deficiencies understaffing and poor care. However, it is unknown whether these experiences are similar for Hispanics with ADRD. We conducted a cross-sectional study using 2016 data from Medicare- certified providers. Our cohort included 1,425,220 short- (30%) and long-term (70%) beneficiaries with ADRD. Approximately 81% of residents were White, 13% African Americans, and 6% Hispanics. African Americans and Hispanics have lower rates of seasonal influenza vaccination (IV). However, they were also less likely to report pain, pressure ulcers and antipsychotic use compared to white residents. After including the facility fixed-effects in the model, it appears that these disparities are mostly due to between-facility level differences.

